# Aspect based sentence segregated dataset of hybrid car's consumers online reviews

**DOI:** 10.1016/j.dib.2022.108293

**Published:** 2022-05-17

**Authors:** Muhammad Faraz Manzoor, Adnan Abid, Naeem A. Nawaz, Atif Alvi

**Affiliations:** a Department of Computer Science, University of Management and Technology, Lahore, Pakistan; b Department of Computer Science, Virtual University of Pakistan, Lahore, Pakistan; c Department of Computer Science, Umm Al-Qura University, Makkah, Saudi Arabia

**Keywords:** Sentiment analysis, Aspects, Natural language processing, Opinion mining

## Abstract

Dataset presented in this paper is obtained from the top online automobile selling and purchasing websites. A total of 1000 reviews related to hybrid cars in the form of text reviews are extracted with the help of the Web Scraper tool. The dataset presents the customers sentiments in the form of reviews related to hybrid cars. Various aspects are taken into consideration while annotating the reviews such as driving, performance, comfort, safety features, interior, exterior and accessories. The annotation of data is done at three levels by three annotators i.e., (1) overall polarity of a review, (2) segregation of the sentence term in which aspect is discussed, (3) polarity of the discussed aspect. Cohen's Kappa score of 0.90 was achieved among the authors while annotating the reviews. Dataset can be used for sentiment analysis, information retrieving, lexicon analysis, and grammatical and morphological analysis.

**Specification Table**

**Table:** 

Subject	Computer Science
Specific Subject Area	Machine Learning, Deep Learning
Type of Data	Text, Reviews
How data were acquired	The web crawler tools are used to gather the data in the raw form from various top websites.
Data Format	Raw, xlsx file
Parameters for data collection	The text data consumer reviews related to automobiles were extracted with the help of the web crawler tool. Positive, negative, and neutral tags were allotted to the reviews using annotation guidelines. The basic text statistics including total review, number of positive reviews, number of negative reviews, number of neutral reviews, minimum and maximum length of review and average length of review were obtained through text complexity analysis.
Data source location	https://www.edmunds.com/, https://www.cars.com/, https://www.autoblog.com/, https://www.pakwheels.com/
Data accessibility	Repository name: Mendeley DataData identification number: 10.17632/k82 × 7czd87.1Direct URL to data: https://data.mendeley.com/datasets/k82 × 7czd87/1

**Value of the Data**

• The dataset of annotated reviews is different from the other traditional review datasets mainly because of the term sentence segregation and its polarity.
• It will facilitate the researchers, manufacturers and marketing agents mainly in the field of automobiles to detect the consumer's experience towards a specific automobile.
• The presented data can be used for various aspect comparison among the automobiles to find out the most discussed aspect.
• The processed dataset will help the automobile buyers to know the aspect wise experience of the automobile owners.

## 1 Data Description

Gathering a dataset is a very challenging task and well-labeled dataset is required to generate accurate results, especially in machine learning-related tasks. There are plenty of datasets available especially for sentiment analysis tasks in various domains such as twitter, movies, mobiles, laptop etc. These datasets show the aspects discussed in the review and overall polarity discussed in the review but the aspect sentence segregation and its context are not discussed in the already discussed dataset. Aspect sentence segregation will help the readers to identify the context in which the aspects are discussed in the review whether it is in a positive manner or a negative manner especially in neutral or conflict review.

There are various information classification techniques that deal with the data collected from various websites and blogs where users post their reviews such as Web-Scraping, Web-Crawling etc. To produce a high-quality dataset to train the classification algorithm, we have gathered the reviews from various popular and highly trending websites such as, edmunds.com, cars.com, autoblog.com and pakwheels.com. Table 1 shows the online websites from which data is gathered for this study. All reviews from these websites are merged as one column in Microsoft Excel 2019. The data added as a pandas Data Frame with the help of ‘read.csv’ function. The formation of excel sheet is such as the review text is followed by the aspect wise review categorization, overall aspects discussed in the review text and review polarity.

**Table 1:** Data collection sources.

Sr#	Website Name	Number of reviews
1	www.Edmunds.com[1]	200
2	www.Cars.com[2]	200
3	www.Autoblog.com[3]	200
4	www.Pakwheels.com[4]	400

### 1.1 Guidelines and Annotation Process

This section discusses the complete procedure that we adopted to annotate the corpus manually. This stage will also define the rules of manual annotation of corpus and calculation of mutual annotator agreement. To add more value and enhance the performance of our corpus, the entire corpus was annotated by the three annotators who are native English speakers. It is pertinent to note that sentiment of reviewers regarding specific car features such as, driving, comfort level, interior, utility and technology are taken into consideration.

In this study Cohen's Kappa Statistic is used to measure the level of agreement between the annotators. Cohen's Kappa score of 0.90 was achieved among the authors using following formula:

(i) $$k\;=\;\left(p{\;}_{o}--\;p_{e}\right)\;/\;\left(1\;--\;p_{e}\right)$$

where:

$p_{o}$: Relative observed agreement among raters.

$p_{e}$: Hypothetical probability of chance agreement.

(1) Positive review guidelines.
(i) If a sentence expressed a positive sentiment for the all the mentioned features of car then it is marked as positive [5].
(ii) If a reviewer writes a review which includes both positive and neutral aspects of a car but positive sentiment trumps the negative review, then it is counted as a positive review [6].
(2) Neutral review guidelines.
(i) Factual information in a sentence makes it a neutral sentence [7].
(ii) If thought is shared in a sentence, then it is classified as neutral [8].
(iii) Sentences with a reduced degree of surety and liability such as words like “maybe” are considered neutral sentences [8].
(iv) A sentence with both positive and negative sentiment in terms of the aspects and entities are classified as a neutral sentence [6].
(3) Negative review guidelines.
(i) If a sentence expressed a negative sentiment for the all the mentioned features of car then it is marked as negative [9].
(ii) Reviews containing more negative terms then positive, then it is counted as negative review [10].
 Sentence including the negation is categorized as negative review.

## 2 Experimental Design, Materials and Methods

**Fig. 1:**
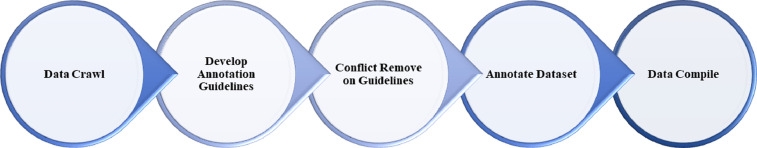
Dataset generation process.

Effective results of any experiment are highly dependent on the quality of the dataset. Therefore, in this research we have defined a well-structured framework to gather and compile data for experimental purposes as shown in Fig. 1.

*Data Gathering:* in the first stage of the data generation, the web crawler tools are used to gather the data in the raw form from various top websites.

*Develop Annotation Guidelines:* To add more value and enhance the performance of our dataset we develop the aspect wise annotation guidelines with the help of the manufacturers from the industry.

*Conflict Remove on Guidelines:* Since two product manufacturers are employed to help in development of annotation guidelines, there is a probability that conflict may occur. Therefore, third annotator will be employed to remove the conflict.

*Dataset Annotation:* Three annotators who are fluent English speakers and are familiar with the sentiment analysis method will perform the annotation to prepare the benchmark opera.

*Data Compile:* The data gathered after annotation may be in different files and in order to process the data easily it has to be merged in an integrated file. Thus, in this stage we perform data integration and merge all the files in Microsoft Excel 2019.

**Fig. 2:**
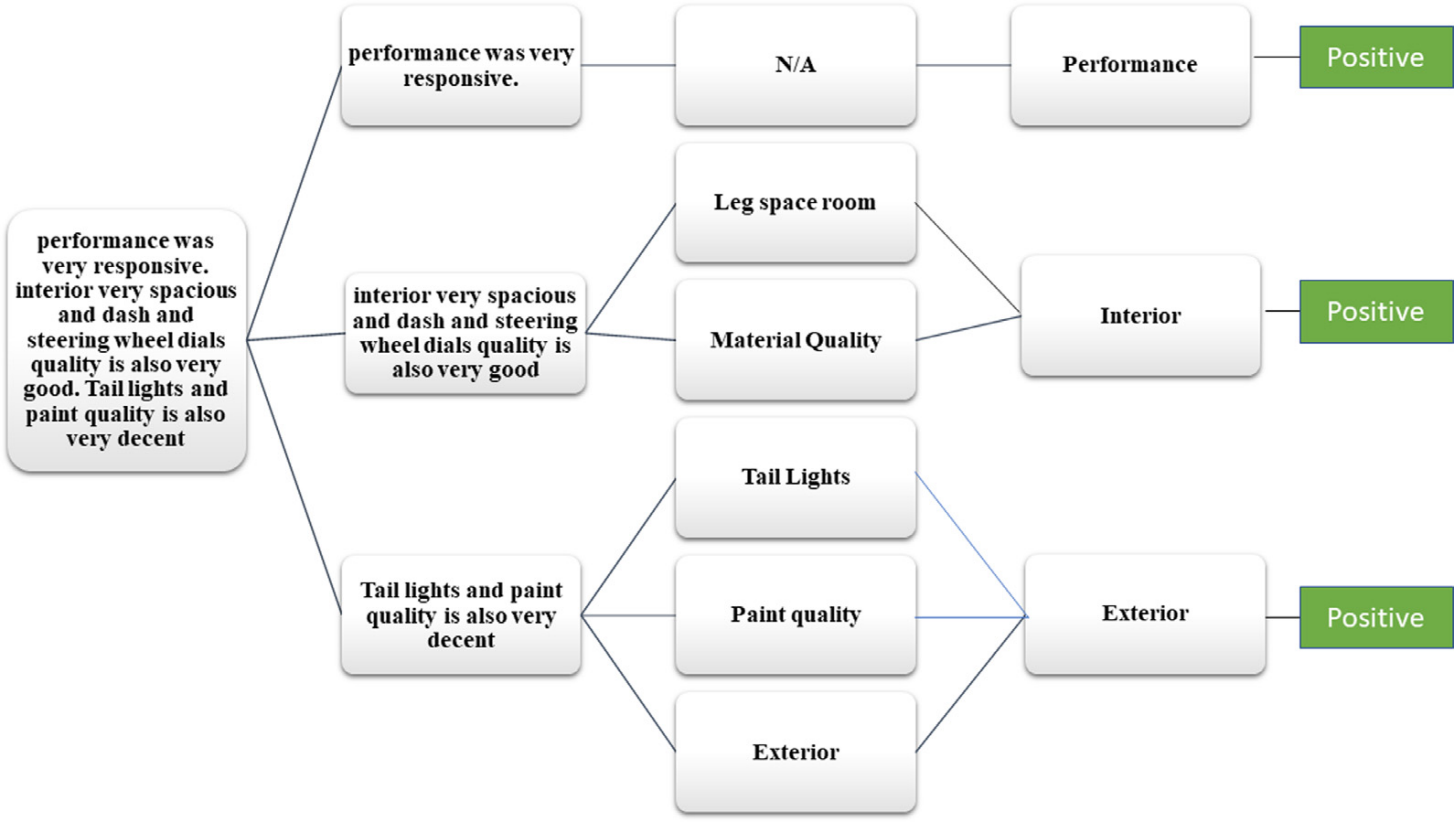
Aspect wise positive review.

**Fig. 3:**
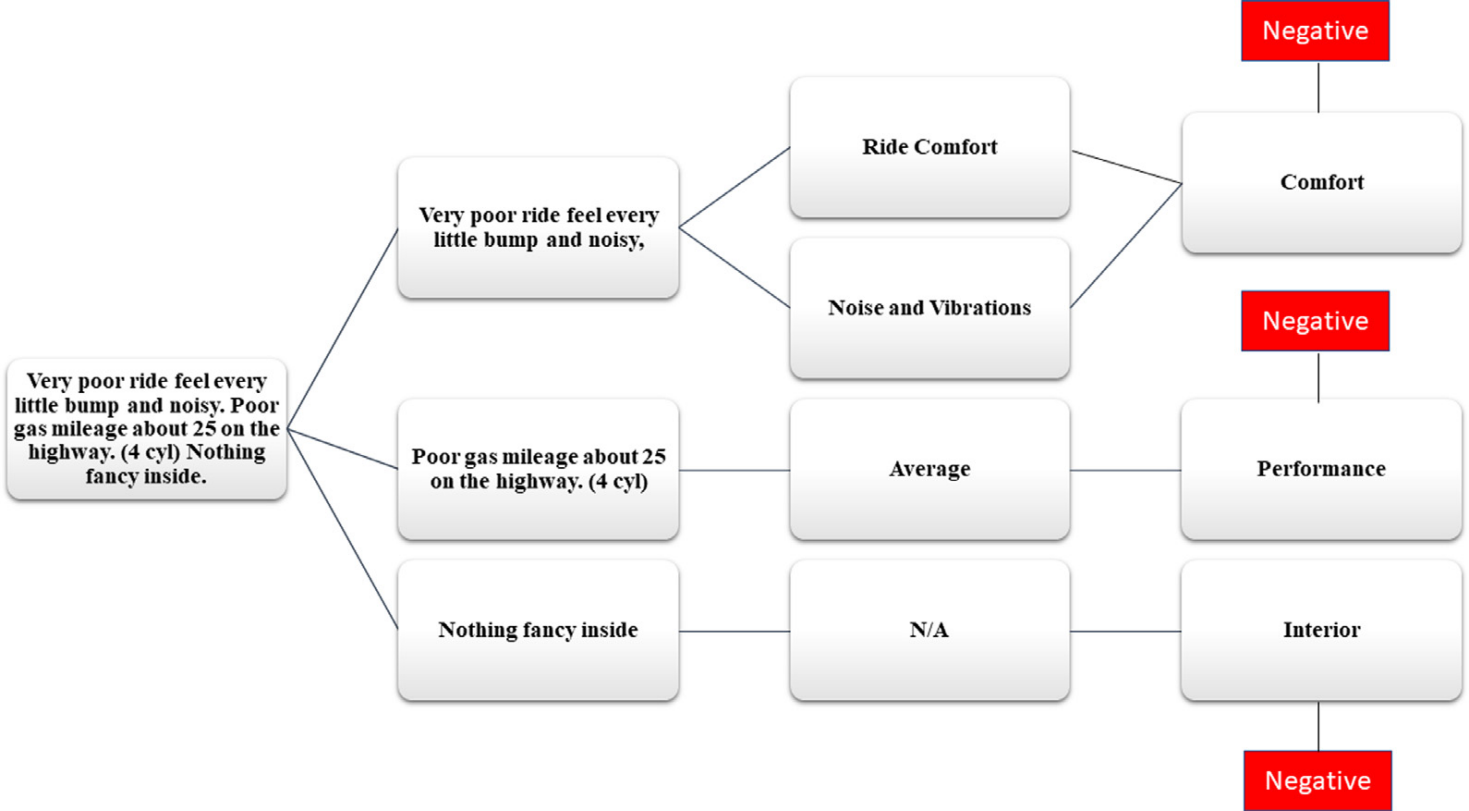
Aspect wise negative review.

**Fig. 4:**
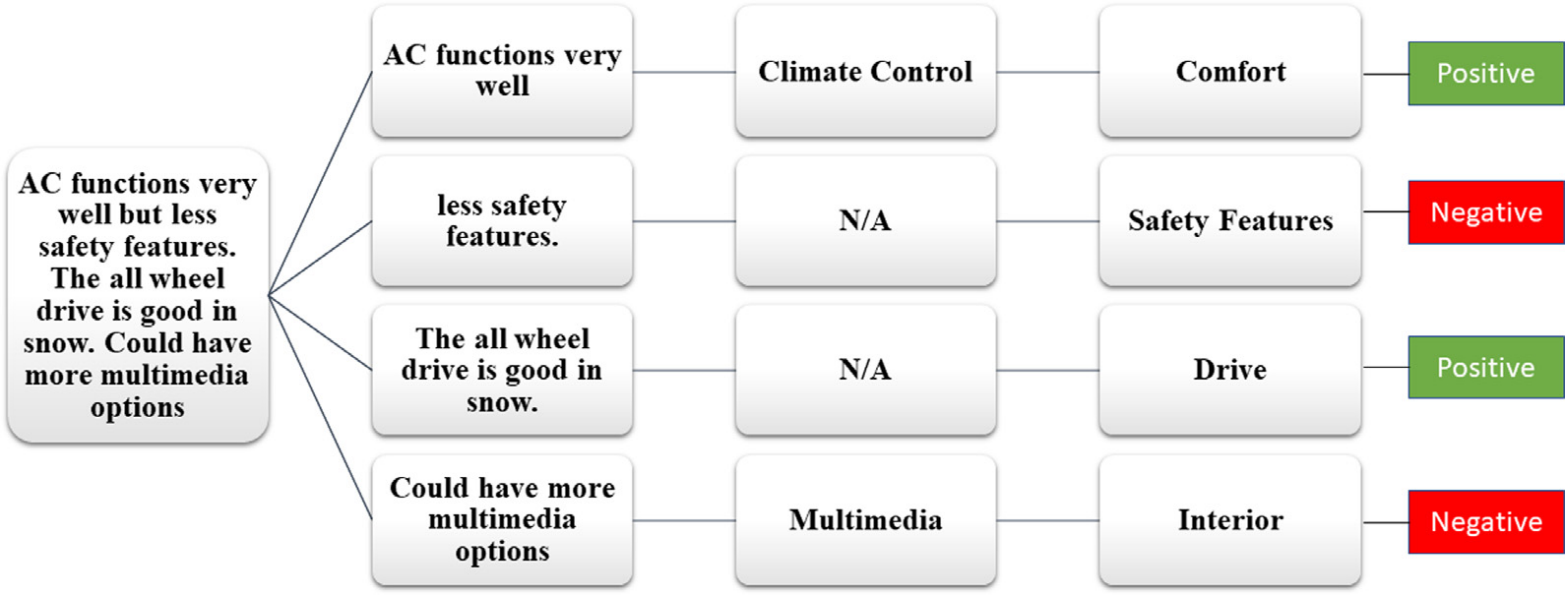
Aspect wise neutral review.

The total 1000 reviews related to hybrid cars in the form of text are extracted with the help of the Web Scraper tool. The dataset presents the customers sentiments in the form of reviews related to hybrid cars. Various aspects are taken into consideration while annotating the reviews such as, driving, performance, comfort, safety features, interior, exterior and accessories. The annotation of data is done at three levels by three authors: (1) overall polarity of a review, (2) segregation of the sentence term in which aspect is discussed, (3) polarity of the discussed aspect. The aspect and sub aspects mapping of the data set is shown in the Table 2. This aspects mapping helps the annotators in categorizing the reviews in positive, negative and neutral categories as shown in Figs. 2,Fig. 3, Fig. 4, respectively.

**Table 2:** Mapping of aspect term and sub aspects of hybrid cars dataset.

Aspect Term	Sub aspects	Aspect Term	Sub aspects
Driving	(i) Acceleration (ii) Braking (iii) Steering (iv) Handling	Exterior	(i) Paint Quality (ii) Head light (iii) Tail light (iv) Ground Clearance (v) Tyre and Rim size (vi) Wipers (vii) Car design
Performance	(i) Engine (ii) Battery and Motors (iii) Torque (iv) Average (v) Mileage	Accessories	(i) Spare tyre (ii) Puncher kit (iii) Air pump (iv) Charging sockets
Comfort	(i) Seat comfort (ii) Ride comfort (iii) Noise and vibration (iv) Suspension (v) Climate control (vi) Room (vii) Doors (viii) Seats control (ix) Heated/Cooling Seats (x) Interior material (Leather, Rexine etc) (xi) Sun Roof (xii) Multimedia controls (steering vs. on dashboard) (xiii) Multimedia connectivity (Bluetooth Vs wire) (xiv) Power Windows (xv) Engine transmission (auto vs. manual) Cruise Control	Safety Features	(i) Air bags (ii) Immobilizer (iii) Seat belts (iv) Child Isofix (v) Braking Technology (vi) Car locks (vii) Lane Guidance (viii) Parking Sensors and Guidance (ix) 360-degree view camera (x) Finger sensors (xi) Car Alerts (on mirror and dashboard) (xii) GPRS (xiii) Tracker
Interior	(i) Head Room space (ii) Leg room space (iii) Material quality (iv) Multimedia (v) Speedometer	Others	Maintenance and its cost Parts availability and the cost Resell value of the car

### 2.1 Statistics of Dataset

**Fig. 5:**
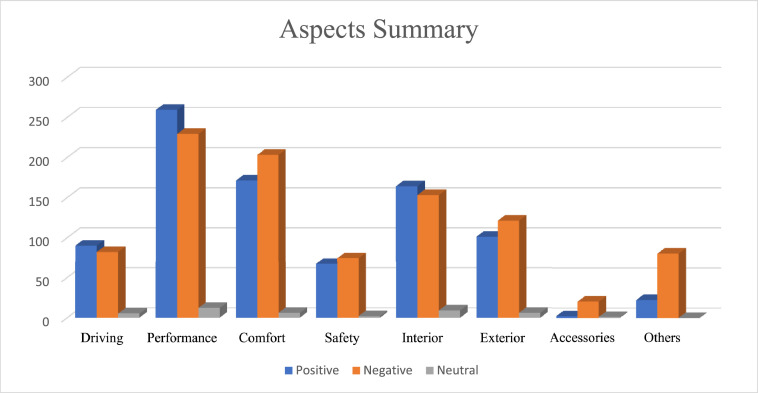
Aspect and category wise statistics.

The overall and aspect wise characteristics of dataset is represented in Table 3 and Fig. 5, respectively. Performance was the most discussed aspect in the review and accessories were the least discussed review in the dataset.

**Table 3:** Characteristics of dataset.

Total positive reviews	406
Total negative reviews	498
Total neutral reviews	96
Minimum length of written review	10 words
Maximum length of written review	347 words
Average length of review	19 words
Total Reviews	1000

## Ethics

(a) Terms of service (ToS): Based on the website ToS, the web resource allowing the data to be scrapped and distributed.
(b) Copyright: The data belonging to the web resource itself.
(c) Privacy: it is recommended to anonymize the data before sharing,
(d) Scrapping policies: There is no such policy.

## CRediT authorship contribution statement

**Muhammad Faraz Manzoor:** Conceptualization, Methodology, Software. **Adnan Abid:** Data curation, Writing – original draft. **Naeem A. Nawaz:** Supervision, Writing – review & editing. **Atif Alvi:** .

## Declaration of Competing Interest

The authors declare that they have no known competing financial interests or personal relationships that could have appeared to influence the work reported in this paper

## Data Availability

Three Level Fully Annotated Car Reviews (Original data) (Mendeley Data).

## Appendix Supplementary materials

Image, application 1
