# Supplementary material for: Aspect based sentence segregated dataset of hybrid car's consumers online reviews
Source: Data Brief. 2022 May 17;42:108293. doi: 10.1016/j.dib.2022.108293 (PMC9142620; doi:10.1016/j.dib.2022.108293)
Supplement: Supplementary file 1 [file mmc1.docx]

Please annotate there following reviews by these tags

1. Positive
2. Negative
3. Neutral

Every Tag have specific column in front of review in excel sheet you just need to put 1 and 0 for selection please don’t put it empty. Also, another section named **Aspect** is added in front the review to categorize the aspects discussed in the review.

Reviews are free text by the customers and users so mistakes such as spellings and grammar are expected. These reviews are specific to cars industry and collected from many renown websites. The common features/aspects of the car in reviews are following:

1. **Driving**
   1. Acceleration
   2. Braking
   3. Steering
   4. Handling
2. **Performance**
   1. Engine
   2. Battery and Motors
   3. Torque
   4. Average
   5. Milage
3. **Comfort**
   1. Seat comfort
   2. Ride comfort
   3. Noise & vibration
   4. Suspension
   5. Climate control
   6. Room
   7. Doors
   8. Seats control
   9. Heated/Cooling Seats
   10. Interior material (Leather, Rexine etc)
   11. Sun Roof
   12. Multi media controls (steering vs. on dashboard)
   13. Multi media connectivity(Bluetooth Vs wire)
   14. Power Windows
   15. Engine transmission (auto vs. manual)
   16. Cruise Control
4. **Safety Features:**
   1. Air bags
   2. Immobilizer
   3. Seat belts
   4. Child Isofix
   5. Braking Technology
   6. Car locks
   7. Lane Guidance
   8. Parking Sensors & Guidance
   9. 360-degree view camera
   10. Finger sensors
   11. Car Alerts (on mirror & dashboard)
   12. GPRS
   13. Tracker
5. **Interior**
   1. Head Room space
   2. Leg room space
   3. Material quality
   4. Multimedia
   5. Speedometer
   6. Any other interior part discussed
6. **Exterior**
   1. Paint Quality
   2. Head light
   3. Tale light
   4. Ground Clearance
   5. Tyre and Rim size
   6. Wipers
   7. **Car design**
   8. Any other exterior part discussed
7. **Accessories**
   1. Extra tyre
   2. Puncher kit
   3. Air pump
   4. Charging sockets
8. **Others**
   1. Maintenance and its cost
   2. Parts availability and the cost
   3. Resell value of the car

Following are some examples to get idea for Tags

Positive Reviews:

**Example**:
“ Love this car, it has plenty of leg and head room for those over six foot. even though it has a small engine its zippy and performs well. Comfortable ride inside for long distance and with the back seats down lots of room for large dogs or anything else you might want to carry. The only down is the side mirrors which are small and take some getting used to.”

Aspects:

Positive: Comfort

Negative: exterior

Negative Reviews:

**Example**
“ I first brought this car in 2018 and two months later the check engine light came on and the car wouldn’t accelerate past the speed I was going and was told #3 times they couldn’t duplicate the problem at the dealership I purchase the car at and the fourth time told me that the problem was a fuel injection need replacing this is far the worst car I ever purchase. ”

Positive:

Negative: Driving

Neutral:

**Example**
“It Is a beautiful car until suddenly it tries to die mid-commute and the transmission is shot. So disappointed. The interior touch screen display was often glitchy as well (and this is the only control for the fan/heat/ac!!). Total lemon and it still even smelled new..”

Positive: Interior/exterior

Negative: Interior
